# Glutathione-responsive disassembly of disulfide dicyanine for tumor imaging with reduction in background signal intensity

**DOI:** 10.7150/thno.39673

**Published:** 2020-01-12

**Authors:** Shanyan Mo, Xiaoting Zhang, Sadaf Hameed, Yiming Zhou, Zhifei Dai

**Affiliations:** 1 Department of Biomedical Engineering, College of Engineering, Peking University, Beijing 100871, China; 2 College of Life Science and Bioengineering, Beijing University of Technology, Beijing 100124, China

**Keywords:** Fluorescence imaging, molecular probe, tumor response, disulfide dicyanine, fluorescence quenching

## Abstract

Near-infrared (NIR) fluorescence imaging has been proved as an effective modality in identifying the tumor border and distinguishing the tumor cells from healthy tissue during the oncological surgery. Developing NIR fluorescent probes with high tumor to background (T/B) signal is essential for the complete debulking of the tumor, which will prolong the survival rate of tumor patients. However, the nonspecific binding and “always-on” properties of the conventional fluorescent probes leads to high background signals and poor specificity.

**Method**: To address this problem, glutathione (GSH)-responsive, two disulfide-bonded dicyanine dyes (**ss-diCy5** and **ss-diNH800CW**) were synthesized. As synthesized dyes are quenched under normal physiological conditions, however, once reached to the tumor site, these dyes are capable of emitting strong fluorescence signals primarily because of the cleavage of the disulfide bond in the tumor microenvironment with high GSH concentration. Besides, the GSH-responsive behavior of these dyes was monitored using the UV-vis and fluorescence spectroscopy. The diagnostic accuracy of the aforementioned dyes was also tested both in tumor cells and 4T1-bearing mice.

**Results**: The fluorescence signal intensity of disulfide dicyanine dyes was quenched up to 89% compared to the mono cyanine dyes, thus providing a very low fluorescence background. However, when the disulfide dicyanine dye reaches the tumor site, the dicyanine is cleaved by GSH into two mono-dyes with high fluorescence strength, thus producing strong fluorescent signals upon excitation. The fluorescent signal of the dicyanine was enhanced by up to 27-fold after interacting with the GSH solution. *In vivo* xenografts tumor studies further revealed that the fluorescence signals of aforementioned dyes can be quickly recovered in the solid tumor.

**Conclusion**: In summary, the disulfide dicyanines dyes can provide a promising platform for specific tumor-activatable fluorescence imaging with improved T/B value.

## Introduction

Fluorescence imaging is an essential means of pathological monitoring and diagnosis at the molecular level 1-9, and the choice of the appropriate fluorescent probe is a key point for this technique 10-19. Conventional fluorescent probes produce fluorescence signals in both cancerous and healthy tissues, which lead to relatively high background signals and poor specificity 20. To address this problem, activatable fluorescent probes have been designed, which is quenched or inactive under normal conditions and showed no or very little fluorescence. However, once reached to the desired tumor site, these activatable probes can emit strong fluorescence signals mainly due to the acidic tumor microenvironment or specific enzyme activity, which improves the T/B ratio and tumor specificity 21.

Cyanine dyes have been broadly explored as always-on probes for fluorescence imaging not only in preclinical but in clinical settings as well, such as CW800 22, ZW800-1 23, Cy5 cDots 24. However, because cyanine dyes are constituted of large conjugate aromatic rings and alkenes, π-π stacking and aggregation are usually prevalent in aqueous solution 25-27. The aggregation nature of these cyanine dyes often causes serious fluorescent quenching problem, thus lowering the signal to noise ratio (SNR) for optical imaging.

Nevertheless, the conjugate 28-33 or non-conjugate 34-37 dicyanine dyes suffer more severe aggregation-caused quenching (ACQ) than the mono cyanine because of the higher short-range molecular interactions such as π-π stacking. However, on the other hand, dicyanines hold the ability to provide extremely low background for fluorescence imaging; and if the fluorescence can be recovered at the tumor site, real-time tumor imaging with high resolution can be easily achieved.

Therefore, attempts to improve the SNR *via* background signal reduction have primarily focused on designing NIR fluorophores whose signals can be modified in response to the local tumor microenvironment (TME) 38-43. For instance, the difference in the intracellular and extracellular concentration of glutathione (GSH) (approximately 2-10 mM in intracellular *vs* 2-20 μM in extracellular) can be realized as an ideal stimulus to improve the SNR of fluorescence dyes. Therefore, the concentration contrasts are broadly harnessed to trigger the cleavage of fluorophores conjugated with a disulfide bond 44-55. This GSH-responsive cleavage enriched the manner of fluorescence signals and offered an efficient approach to enhance the SNR of bioimaging.

Herein, we report the synthesis of disulfide-bonded dicyanines, including a disulfide Cy5 compound (**ss-diCy5**) and a hydrophilic disulfide amine substituted 800CW compound (**ss-diNH800CW**). The results show that the fluorescence strength is quenched by 55% for **ss-diCy5** and 89% for **ss-diNH800CW** in PBS compared to the corresponding mono cyanine. After treatment with GSH solution, the fluorescence is recovered for **ss-diCy5**; and a new fluorescent peak at 798 nm is generated for **ss-diNH800CW**. *In vitro* studies revealed that the fluorescence of disulfide-bonded dicyanines could be turned on by the breaking of the disulfide bond in cancer cells. *In vivo* and *ex vivo* tumor studies revealed that the fluorescence could be recovered in the solid tumor. This approach represents a significant advantage of specific tumor-activatable fluorescence imaging with improved T/B value.

## Materials and Methods

The chemicals used in the article were purchased from Innochem, Sigma-Aldrich, TCI, Fluka, Alfa Aesar or purchased from the Bio&Chem Reagent Management Plat in PKU. Commercial reagents and solvents were obtained in >97% purity and used directly. Mass spectrometry was performed on an AB Sciex 5800 MALDI-TOF/TOF Mass Spectrometer or a Bruker Solarix XR Fourier Transform Ion Cyclotron Resonance Mass Spectrometer. ^1^H and ^13^C NMR spectra were recorded on Bruker Avance (500 MHz) spectrometer. Vis/NIR absorption spectra were measured on a UV-vis spectrophotometer (Varian 4000). Fluorescence spectra were recorded using a fluorescence spectrophotometer (Thermo Fisher Scientific Lumina).

### Synthesis of c-diCy5

The solution of dihydrazine compounds (3 g) in AcOH (20 mL) was added 3-methylbutanone (5.15 g) and heated to reflux for 12 hours. Then AcOH was removed and afforded the crude diindole intermediate, which was directly used for the next step.

^1^H NMR (500 MHz, CDCl_3_) *δ* 7.44 (d, *J* = 7.8 Hz, 2H), 7.12 (dd, *J* = 7.8, 1.6 Hz, 2H), 7.09 (d, *J* = 1.6 Hz, 2H), 4.06 (s, 2H), 2.26 (s, 6H), 1.27 (s, 12H).

The crude product of diindole (1 g) was added 1,4-butane sultone (2.66 g). The mixture was heated to 120 ^o^C and kept on stirring for 3 hours, then cooled down to ambient temperature. A purple solid was formed, which was washed three times with acetone and dried under vacuum. The methylene diindole quaternary ammonium salt was obtained as pink solid. MS (MALDI-TOF): *m/z* = 603.26, calcd. for C_31_H_43_N_2_O_6_S_2_ ([M+H]^+^) = 603.25. ^1^H NMR (500 MHz, D_2_O) *δ* 7.62 (d, *J* = 8.4 Hz, 2H), 7.54 (d, *J* = 1.2 Hz, 2H), 7.40 (dd, *J* = 8.4, 1.2 Hz, 2H), 4.39 (t, *J* = 7.6 Hz, 4H), 4.13 (s, 2H), 2.84 (t, *J* = 7.5 Hz, 4H), 2.04 - 1.92 (m, 4H), 1.82 - 1.68 (m, 4H), 1.42 (s, 12H).

**c-DiCy5**. Methylene diindole quaternary ammonium salt (50 mg) and malondialdehyde dianil hydrochloride (45 mg) was refluxed in a solution of AcOH/Ac_2_O (2 mL, 1:1 ratio) for 2 hours. Then the hemicyanine intermediate precipitated by addition of Et_2_O and dried under vacuum. The intermediate was re-dissolved in AcOH (1 mL), adding 4-sulfobutyl quaternary ammonium indole (61.7 mg) and pyridine (1 mL). The solution was heated to 60 ^o^C for 2 hours. The product was precipitated as green solid and washed 3 times with Et_2_O. The solid was purified with a reversed-phase C18 column utilizing 5% to 35% gradient of MeOH in H_2_O to afford the pure **c-diCy5** (62%). HRMS (MALDI-TOF): *m/z* = 1381.52073, C_71_H_89_N_4_O_16_S_4_ calcd. for [M+H]^+^ = 1381.51509. ^1^H NMR (500 MHz, DMSO) δ 9.06 (s, 2H), 8.43 - 8.31 (m, 4H), 7.84 (d, *J* = 1.2 Hz, 2H), 7.71 (s, 4H), 7.69 - 7.66 (m, 2H), 7.45 (d, *J* = 8.3 Hz, 2H), 7.38 - 7.30 (m, 4H), 6.64 (t, *J* = 12.3 Hz, 2H), 6.46 (d, *J* = 13.9 Hz, 2H), 6.31 (d, *J* = 13.6 Hz, 4H), 4.14 (d, *J* = 15.7 Hz, 10H), 1.84 (d, *J* = 6.2 Hz, 4H), 1.80 - 1.68 (m, 36H), 1.65 - 1.56 (m, 4H), 1.47 - 1.37 (m, 4H).

### Synthesis of ss-diCy5

To a suspension of the 6-carboxylic hexyl quaternary ammonium indole (100 mg) and TSTU (104 mg) in 1 mL of dry DMF was added DIPEA (114 mg) and stirred at 25 ^o^C for 0.5 hour. Then cystamine dihydrochloride (43 mg) in 50 μL of DI water was added in one-portion to the solution. After stirring for 5 hours, Et_2_O was added to precipitate the product. The solid was purified on a reversed-phase C18 column using 5% to 45% gradient of MeOH in H_2_O to afford diindole quaternary ammonium salt as colorless solid (81%). MS (MALDI-TOF): *m/z* = 795.30, calcd. for C_36_H_51_N_4_O_8_S_4_^+^ ([M+H]^+^) = 795.26. ^1^H NMR (400 MHz, D_2_O) *δ* 7.96 - 7.76 (m, 2H), 7.76 - 7.52 (m, 2H), 6.55 (d, *J* = 8.5 Hz, 2H), 3.69 - 3.52 (m, 4H), 3.44 - 3.31 (m, 2H), 3.23 - 3.14 (m, 2H), 3.15 - 3.03 (m, 4H), 2.96 - 2.81 (m, 4H), 1.71 (s, 8H), 1.28 - 1.17 (m, 14H), 0.99 (s, 4H).

Diindole quaternary ammonium salt (15 mg) and malondialdehyde dianil hydrochloride (9.3 mg) was refluxed in a solution of AcOH/Ac_2_O (1 mL, 1:1 ratio) for 2 hours. Then the hemicyanine intermediate precipitated by addition of Et_2_O and dried under vacuum. The intermediate was re-dissolved in AcOH (0.5 mL), adding 4-sulfobutyl quaternary ammonium indole (15.6 mg) and pyridine (0.5 mL). The solution was heated to 80 ^o^C for 2 hours. The product was precipitated with Et_2_O as blue solid, and washed 3 times with Et_2_O. The blue precipitate was purified on a reversed-phase C18 column using 5% to 35% gradient of MeOH in H_2_O to afford **ss-diCy5** (63%).

HRMS (MALDI-TOF): *m/z* = 1617.42211, C_72_H_93_N_6_O_20_S_8_ (calcd. for [M+H]^+^ = 1617.42048.^1^H NMR (400 MHz, D_2_O) *δ* 8.68 (d, *J* = 5.4 Hz, 1H), 8.53 (t, *J* = 7.4 Hz, 1H), 7.96 (m, 2H), 7.86 - 7.49 (m, 9H), 7.33 - 7.13 (m, 3H), 6.45 - 6.26 (m, 2H), 6.09 (d, *J* = 13.4 Hz, 3H), 4.43 (s, 2H), 4.03 (m, 5H), 3.71 - 3.45 (m, 4H), 3.35 (s, 4H), 3.11 - 2.99 (m, 2H), 2.93 - 2.79 (m, 8H), 2.63 (m, 4H), 2.72 - 2.33 (m, 4H), 1.89 (m, 10H), 1.51 - 1.11 (m, 14H), 1.30 - 1.11 (m, 8H).

### Synthesis of ss-diNH800CW

The cystamine dihydrochloride (22.2 mg), **Cl-800CW** (200 mg) and Na_2_CO_3_ (22.3 mg) was added to 2 mL DI water. After heating to 50 ^o^C for overnight, the solution was cooled to 25 ^o^C and purified on a reversed-phase C18 column using 1% to 25% gradient of MeOH in H_2_O to afford **ss-diNH800CW** (72%) and **ss- mono NH800CW** (10%). **ss-diNH800CW:** HRMS (MALDI-TOF): *m/z* = 1853.44022, C_80_H_105_N_6_O_24_S_10_ (calcd. for [M+H]^+^ = 1853.43818. ^1^H NMR (500 MHz, D_2_O) *δ* 7.86 - 7.57 (m, 12H), 7.15 - 6.92 (m, 4H), 5.84 - 5.64 (m, 4H), 4.15 - 3.93 (m, 4H), 3.50 (s, 5H), 3.00 - 2.88 (m, 5H), 2.88 - 2.73 (m, 8H), 2.49 - 2.28 (m, 8H), 1.81 - 1.61 (m, 22H), 1.57 (s, 24H). **ss- mono NH800CW**: HRMS (MALDI-TOF): *m/z* = 1003.24264, C_42_H_59_N_4_O_12_S_6_ (calcd. for [M+H]^+^ = 1003.24482 (Figure S12; Supplementary Information).

### Cell Culture and Cell Uptake Studies

4T1 murine breast cancer cells were cultured in DMEM medium (Gibco, USA) supplemented with 10% fetal bovine serum (BI, USA) and 1% penicillin/streptomycin. The cellular uptake and intracellular localization of **ss-diCy5**, **c-diCy5** and **mono-Cy5** were observed by a fluorescence microscopy. 4T1 cells were seeded on 24-well plate at a density of 5x10^4^ per well and incubated overnight. Then the pre-seeded cells were exposed to **ss-diCy5** (5 μM), **mono-Cy5** (10 μM) or **c-diCy5** (5 μM) for 4 h. After washing with PBS for 3 times, the intracellular fluorescence of Cy5 was imaged with a fluorescence microscope (Leica DMI3000B, Wetzlar, Germany; ×400 magnification).

### In Vivo Fluorescence Imaging Studies

The animal experiment was approved by Peking University Institutional Animal Care and Use Committee (IACUC). To prepare tumor-bearing mice model, a volume of 50 μL cell suspension containing 1 × 10^7^ 4T1 cells was subcutaneously injected into the right flanks of female Balb/C mice. For in vivo fluorescent imaging, **ss-diCy5**, **c-diCy5** at a dose of 0.05 μmol kg^-1^, and **mono-Cy5** at a dose of 0.1 μmol kg^-1^ was injected intratumorly. Then whole-body fluorescence imaging was obtained with an IVIS Imaging Spectrum System (Perkin Elmer) at different time points after injection. Cy5 fluorescence signals were excited by 620 nm and collected from 670 nm. **ss-diNH800CW** at a dose of 0.05 μmol kg^-1^, and **mono-ssNH800CW** at a dose of 0.1 μmol kg^-1^ was injected intratumorly. 800CW fluorescence signals were excited by 780 nm and collected from 825 nm.

### In Vitro Cytotoxicity and In Vivo Biocompatibility Studies

Cytotoxicity of the various ss-didyes was evaluated in 4T1 cells. 4T1 cells were pre-seeded in 96-well at a density of 5,000 per well and cultured for 4 h, followed by incubation with various concentrations of **mono-Cy5**, **ss-diCy5**, **c-diCy5**, **ss-diNH800CW** and **mono-NH800CW**. 24 h later, methyl thiazolyl tetrazolium (MTT) assay was used to evaluate the cell viability.

For biocompatibility studies, the mice were randomly divided into 6 groups (n = 3 per group). The 6 groups of mice were intravenously injected with **ss-diCy5**, **c-diCy5**, **ss-diNH800CW** at a dose of 0.5 µmol kg^-1^, **mono-Cy5**, **mono-NH800CW** at a dose of 1 µmol kg^-1^, or PBS. After various injection, the body weights were recorded every day for 14 days. Then mice were sacrificed and main organs (heart, liver, spleen, lung and kidney) were excised for H&E staining to evaluate the biocompatibility of various ss-didyes. The H&E images were obtained with a microscope (Leica DMI3000B, Wetzlar, Germany).

## Results and Discussion

### Synthesis

The synthesis of mono cyanine dyes **mono-Cy5** 56, **Cl-800CW** 57 was accomplished according to the already reported method. As depicted in Figure **S1**, the dicyanine **ss-diCy5** was synthesized via a two-step route: first, two equivalent of 6-carboxylic hexyl quaternary ammonium indole (**1**) reacted with one equivalent of cystamine to produce diindole quaternary ammonium salt(**2**) in the presence of TSTU and DIPEA; second, the diindole quaternary ammonium salt (**2**) was condensed with malondialdehyde dianilhydrochloride to produce the hemicyanine intermediate then reacts with 4-sulfobutyl quaternary ammonium indole(**3**) in the presence of pyridine to generate **ss-diCy5**.

The dicyanine with methylene bond **c-diCy5** was synthesized via a two-step pathway (Figure **S2**): first, condensation of methylene diindole quaternary ammonium salt (**4**) with malondialdehyde dianilhydrochloride in the presence of acetic anhydride produce the hemicyanine intermediate; then the intermediate reacts with quaternary ammonium indole(**5**) to obtain **c-diCy5**. The hydrophilic disulfide amine substituted 800CW compound (**ss-diNH800CW**) was synthesized via nucleophilic substitution of chlorine fluorophore **Cl-800CW** with cystamine (**Figure S11**, Supporting Information). Meanwhile, the reaction produces **ss-monoNH800CW** as a by-product.

### Comparison of the optical properties of mono cyanine dyes and dicyanine dyes

**Figure 1:**
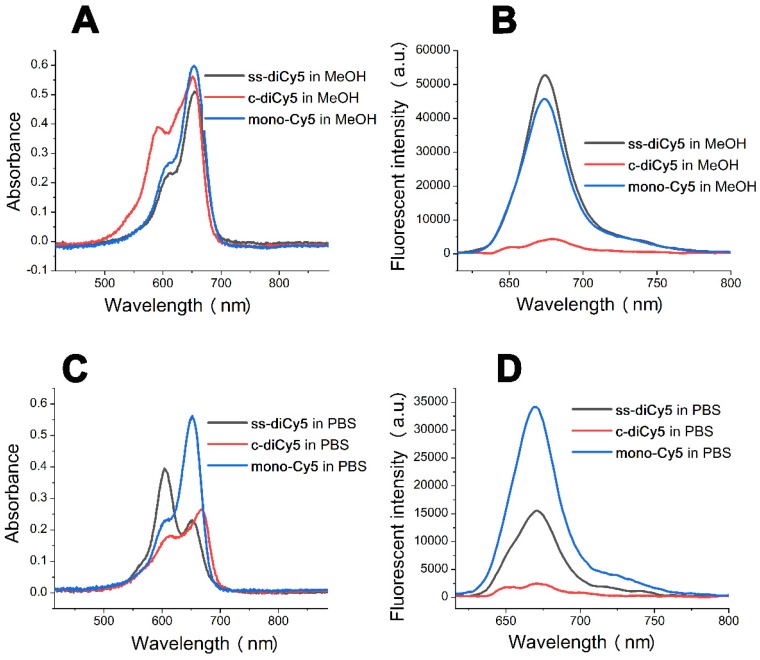
The absorption spectra of the dyes **ss-diCy5** (5 μM), **c-diCy5** (5 μM) and **mono-Cy5** (10 μM) in MeOH (A) and PBS (C) and fluorescent spectra in MeOH (B) and PBS (D). Inset B and D: *λ_ex_* = 650 nm

As synthesized three Cy5 dyes, **mono-Cy5** (10 μM), **ss-diCy5** (5 μM), and **c-diCy5** (5 μM) were dissolved in methanol and PBS, respectively and their ultraviolet-visible (UV-vis) absorption spectrum and fluorescence spectrum were observed as shown in Figure 1.

Figure 1A indicates that in methanol, the absorbance peaks of all three Cy5 dyes were similar at around 650 nm with similar intensity. A clear shoulder peak appeared at 590 nm in the UV-vis absorption spectra of **c-diCy5**, indicating the aggregation phenomenon in the solution. Fluorescence spectrum of **c-diCy5** further validated the presence of aggregation in the solution as the fluorescence intensity of **c-diCy5** is only ~ 9.6% of that of **mono-Cy5**. However, the fluorescence intensity of **ss-diCy5** was almost equivalent to that of **mono-Cy5**, denoting minimal aggregation in the **ss-diCy5** methanol solution (Figure 1B). Since methanol is an excellent solvent for all three Cy5 dyes, intermolecular aggregation is negligible in the methanol solutions. These observations highlighted that the intramolecular interaction in the **c-diCy5** is mainly responsible for the aggregation phenomenon.

In the UV-vis absorption spectrum measured in PBS (Figure 1C), a clear shoulder peak at around 590 nm appeared with a much higher intensity than the 650 nm peak in the **ss-diCy5** solution, while the spectra of **c-diCy5** in PBS is similar with that in methanol. Moreover, the peak at 650 nm of both of the dicyanine Cy5 dyes is much lower that of **mono-Cy5**, indicating the aggregation of both dicyanine dyes in PBS solution. This is largely due to the presence of intermolecular aggregation of the **ss-diCy5** and both intermolecular and intramolecular aggregation of **c-diCy5** in PBS, which leads to the fluorescence intensity quenching by 55.0% for **ss-diCy5** and 92.8% for **c-diCy5** compared to the intensity of **mono-Cy5** (Figure 1D).

Thus, the self-aggregation of dicyanine dyes is more severe in PBS. This is primarily due to the fact that the two cyanines are one carbon atom apart in the **c-diCy5** molecule and a total of ten atoms apart in the **ss-diCy5** molecule, thereby, a conclusion can be drawn that the closer the two cyanines in the dicyanine molecule are, the stronger the aggregation in the PBS solution will be, and the more fluorescence quenching will be observed.

**Figure 2:**
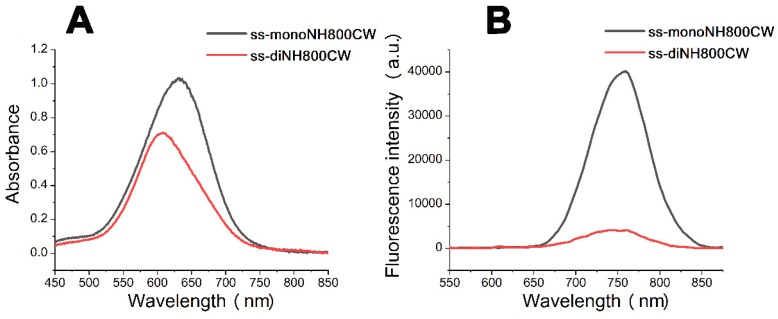
The absorption spectra (A) and fluorescent spectra (B) of the dyes **ss-diNH800CW** (5 μM) and **ss-monoNH800CW** (10 μM) in PBS. Inset B: *λ_ex_* = 608 nm

In order to further verify this hypothesis, we next synthesized another disulfide dicyanine, named **ss-diNH800CW**. The obtained two NH800CW dyes **ss-monoNH800CW** (10 μM)**, ss-diNH800CW** (5 μM) were dissolved in PBS, and their ultraviolet-visible absorption spectrum and fluorescence spectrum were studied, which were shown in Figure 2. These results revealed a blue shift from 630 nm to 608 nm in the absorption band of **ss-diNH800CW**, while the fluorescence spectrum showed that the fluorescence intensity of **ss-diNH800CW** was only 11% of the intensity of the **ss-monoNH800CW**. Besides, in the dark condition, the absorbance intensity reduced less than 10% after 36 hours at the pH of both 7.4 and 6.0, and the fluorescence intensity remained unchanged, suggesting high stability of dyes in the dark. Thus, the diNH800CW dyes can offer an excellent fluorescence background for bio-imaging.

### Fluorescence recovering of ss-didyes upon the addition of GSH

**Figure 3:**
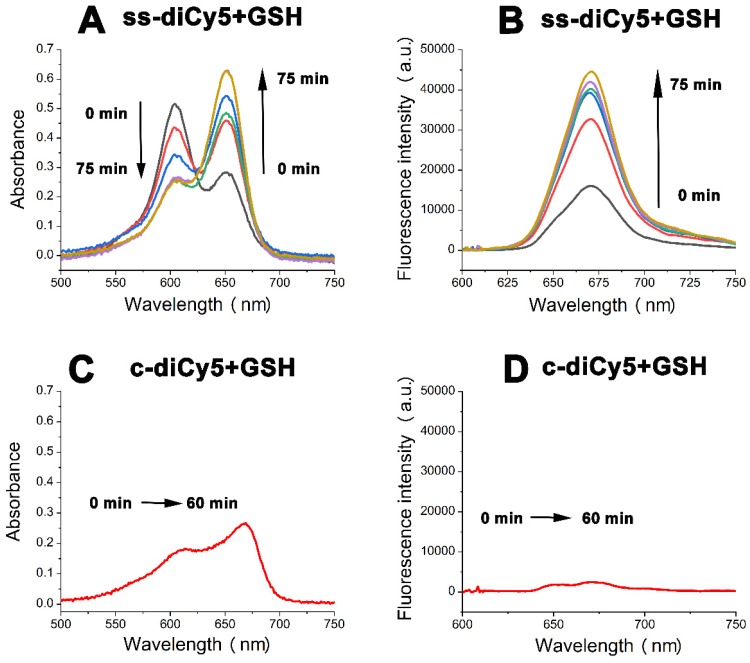
The absorption spectra (A and C) and fluorescent spectra (B and D) changes of **ss-diCy5** (5 μM) and **c-diCy5** (5 μM) in the presence of GSH (1.5 mM) in PBS solution (pH = 7.4, 25 ^o^C). Inset B and D: *λ_ex_* = 650 nm

The fluorescence quenching property of disulfide dicyanine dyes provides an excellent background; however, in order to use the dicyanine dyes in optical imaging, the fluorescent strength needs to be enhanced at the tumor site. Therefore, we investigated the spectral properties of disulfide dicyanine dyes in the presence of GSH. As anticipated, changes in absorption and fluorescence spectrum of the disulfide dicyanine dye were observed after treating with GSH (1.5 mM) in the PBS solution (pH = 7.4, 5 mM). After treating with GSH, the absorption band of 590 nm decreased while the absorption band of 650 nm increased (Figure 3A); meanwhile, the fluorescence intensity enhanced by about three-folds (Figure 3B). On the contrary, the spectral properties of c-diCy5, the control disulfide dicyanine **Cy5** with methylene bond (“CH_2_”) as a linker, remained unchanged after treating with GSH (Figure 3C and 3D).** ss-diCy5** and **c-diCy5** revealed explicit spectral changes upon reaction with GSH, which is mainly due to the cleavage of disulfide linker of ss-diCy5.

To collect more evidence, the **ss-diCy5** GSH solution was subjected to MALDI-TOF MS analysis. After interaction of GSH (1.5 μM) with **ss-diCy5** (5 mM) for 30 minutes, peaks of 1116.68 (corresponding to [s-Cy5 + GSH + H]^+^) and 810.67 (corresponding to [s-Cy5+ H]^+^) were observed simultaneously in the MALDI-TOF MS spectra (MS, Figure S13; Supplementary Information), demonstrating the cleavage of disulfide linker and formation of new mono Cy5 dyes with strong fluorescence intensity.

**Figure 4:**
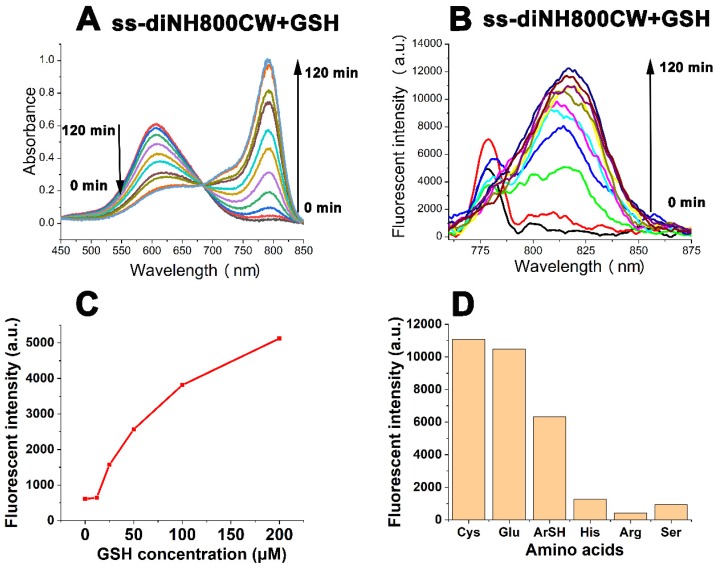
The absorption spectra (A) and fluorescent spectra (B) changes of **ss-diNH800CW** (5 μM) in the presence of GSH (1.5 mM) in PBS solution (pH = 7.4, 25 ^o^C). Fluorescence response of **ss-diNH800CW** (5 μM) with the addition of 25 μM to 200 μM of GSH (C) and 2 mM of amino acids (D) in PBS (pH = 7.4, 25 ^o^C). The data were recorded after 30 mins of the addition at room temperature. Inset B and C: *λ_ex_* = 780 nm

Next, the spectral properties of **ss-diNH800CW** upon reaction with GSH were studied. After treatment with GSH, the absorption band of 608 nm gradually decreased in the **ss-diNH800CW** solution as a new band appeared and enhanced at 780 nm instead of 630 nm (Figure 4A), and the fluorescence signal strength at 798 nm enhanced by 27-fold (Figure 4B). After addition of the GSH for 120 minutes, the band of 608 nm almost disappeared, and the band of 780 nm reached maximum intensity. It was assumed that the** ss-diNH800CW** first reacted with GSH to form the disulfide intermediates GSH-s-NH800CW, then GSH-s-NH800CW quickly continued reacting with the excess of GSH to generate the thiol nucleophilic substitution product GSH-NH800CW. (MS, Figure S14; Supplementary Information) The mass spectroscopy result of the **ss-diNH800CW** GSH solution proved that the dicyanine dye **ss-diNH800CW** was completely converted into GSH-NH800CW (peak of 1186.87, corresponding to [GSH-NH800cw + Na]^+^) (MS, Figure S13; Supplementary Information).

To further explore the interaction of **ss-diNH800CW** with GSH, a series of experiments to observe the change in the fluorescence response of **ss-diNH800CW** upon the addition of GSH solution (25 μM to 200 μM). Results showed that the fluorescence recovery of the disulfide bonded dicyanine was closely related to the concentrations of GSH.(Figure 4C) In addition, the solutions of **ss-diNH800CW** were treated with different amino acids, including cysteine(Cys), glutathione(Glu), 4-mercaptobenzoic acid(ArSH), histidine(His), arginine(Arg), serine(Ser). The results manifested the rapid fluorescence recovery of the disulfide bonded dicyanine with the additions of thiol-containing compounds(Cys, Glu, ArSH), while little fluorescence recovery of the disulfide bonded dicyanine were achieved with the compounds without thiol containing structures(His, Arg, Ser) (Figure 4D). This phenomenon was in line with the results already reported literature 38. Besides, considering the acidic environment of the tumor tissue, the effect of pH on the fluorescence recovery of **ss-diNH800CW** was investigated.^58^ The results demonstrated that the fluorescence intensity of ss-diNH800CW was enhanced slower and fewer at a pH of 6.0 than that at a pH of 7.4. This is because at the acidic environment, the nucleophilic ability of the SH group in GSH was decreased, leads to the reduction in the speed and effectiveness of the nucleophilic reaction and thus the enhancement of the fluorescence intensity was slower and fewer. Nevertheless, the fluorescence intensity of **ss-diNH800CW** was enhanced by 24-fold at the pH of 6.0 after 6 hours of the addition of GSH. (Figure S16; Supplementary Information) Thus, GSH showed great potential for the fluorescence recovery of the disulfide dicyanines, even in the acidic tumor microenvironment.

### Living Cell Imaging

**Figure 5:**
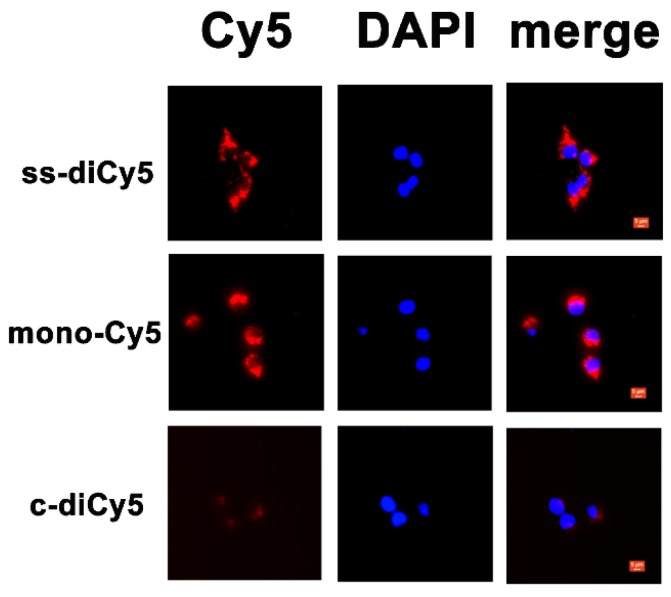
Cellular uptake of **ss-diCy5**, **c-diCy5** and **mono-Cy5**. Scale bar = 5 μm. 4T1 cells were seeded at the density of 5x104 per well on 24-well plate and cultured overnight. Then the pre-seeded cells were incubated with ss-diCy5, mono-Cy5 or c-diCy5 at a Cy5 equivalent concentration of 10 μM. After that, the cells were incubated for 4 h. Finally, the intracellular fluorescence of Cy5 was tested with a fluorescence microscope (Leica DMI3000B, Wetzlar, Germany; ×400 magnification).

As a next step, the resulted disulfide dicyanines were evaluated for the enhanced release of mono-dye in the presence of GSH *in vitro*. The cellular uptake and intracellular localization of **ss-diCy5**, **c-diCy5,** and **mono-Cy5** were examined by fluorescence microscopy. The fluorescence from **ss-diCy5** and** mono-Cy5** (red) was observed in the cytoplasm, suggesting that the **ss-diCy5** was effectively taken up by cancerous cells and transformed into mono dye in the presence of intracellular GSH. (Figure 5) In contrast, the failure to observe the fluorescence of **c-diCy5** *in vitro* indicated the high stability of methylene linkage in the **c-diCy5** molecule. Therefore, the activatable dye **ss-diCy5** with fluorescence switch can be used as an ideal probe for the fluorescence imaging of cancer cells.

### Biocompatibility

**Figure 6:**
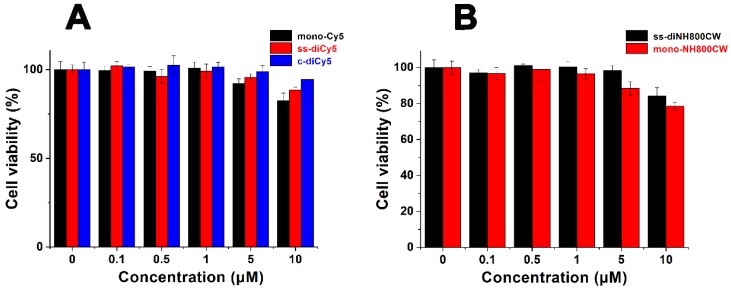
Cell viability of 4T1 cells treated by various concentration of (A) **mono-Cy5**, **ss-diCy5**, **c-diCy5** and (B) **ss-diNH800CW**, **mono-NH800CW**. 4T1 cells were pre-seeded in 96-well at a density of 5,000 per well and cultured for 4 h, followed by incubation with various concentrations of **mono-Cy5**, **ss-diCy5**, **c-diCy5**, **ss-diNH800CW** and **mono-NH800CW**. 24 h later, MTT assay was used to evaluate the cell viability.

The *in vitro* cytotoxicity of ss-didyes was evaluated by MTT assay. 4T1 cells were treated with different concentrations of **ss-diCy5**, **c-diCy5**, **mono-Cy5** and **ss-diNH800CW**, **ss-monoNH800CW**. As shown in Figure 6, the increase in the concentration of ss-didyes only led to a slight decrease in cell viability, indicating the excellent biocompatibility of **ss-diCy5** and** ss-diNH800CW** *in vitro*.

### *In vivo* imaging

**Figure 7:**
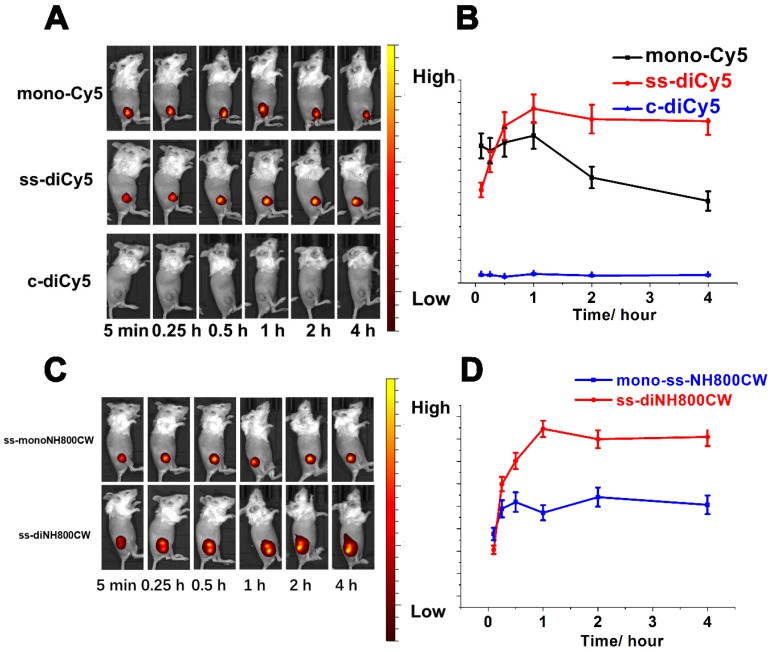
*In vivo* bioimaging picture (A) and fluorescent signals strength changes (B) of tumor-bearing mice at various time (5 min, 15 min, 30 min, 1 h, 2 h, 4 h after intratumorly injection of **ss-diCy5**, **c-diCy5** at a dose of 0.05 μmol kg^-1^, and **mono-Cy5** at a dose of 0.1 μmol kg^-1^. *λ_ex_* = 620 nm, *λ_em_*: 670 nm. *In vivo* bioimaging picture (C) and fluorescent signals strength changes (D) of tumor-bearing mice at various time (5 min, 15 min, 30 min, 1 h, 2 h, 4 h after intratumorly injection of **ss-diNH800CW** at a dose of 0.05 μmol kg^-1^, and **mono-ssNH800CW** at a dose of 0.1 μmol kg^-1^. *λ_ex_* = 780 nm, *λ_em_*: 831 nm.

The satisfying results obtained from *in vitro* studies inspired us to conduct the feasibility research of disulfide dicyanines as an *in vivo* NIR fluorescence dye. The *in vivo* fluorescent recovering performance of the synthetic dyes was studied with tumor-bearing mice. The dyes were injected intratumorally, and the fluorescence images were acquired with a small-animal imaging system. Among the Cy5 dyes, nearly 5 min after the intratumor injection of **ss-diCy5** and **mono-Cy5**, the fluorescence signals were distinctly observed; meanwhile, almost no fluorescence was observed after injection of **c-diCy5**. The fluorescence kept heightening after the injection of **ss-diCy5**, reached the maximum at 1 hour, and lasted for more than 3 hours. (Figure 7A and 7B) The same tendency was observed for the NH800CW dyes. (Figure 7C and 7D) For **ss-diNH800CW**, the fluorescence enhancement was more pronounced. The fluorescence signals were produced quickly inside the tumor and last for a few hours. Intriguingly, the fluorescence intensity of both **ss-diCy5** and **ss-diNH800CW** was higher than their corresponding mono dyes.

However, the fluorescence imaging results of intravenous injection of the disulfide dicyanine dyes were quite disappointing. The dicyanine dyes were cleared very quickly through kidneys. Even 24 h after the intravenous injection, the fluorescence intensity in the liver and spleen were much higher than that accumulated into the tumor, suggesting either the targeted modification of the disulfide dicyanine dyes or assembly into nanoparticles are necessary for the optical imaging of the tumor tissue.

### In Vivo Biocompatibility

**Figure 8:**
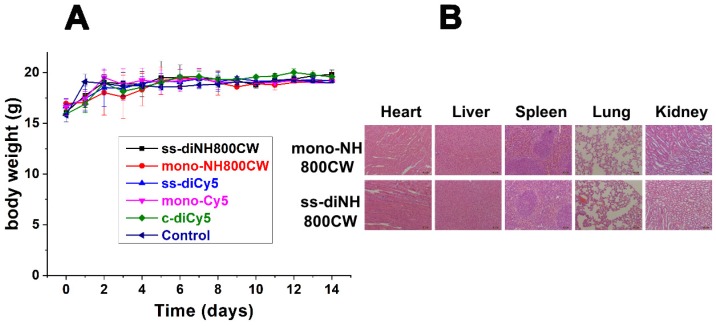
(A) Body weight of different groups of mice after intravenously injected with **ss-diCy5**, **c-diCy5**, **ss-diNH800CW** at a dose of 0.5 μmol kg^-1^, **mono-Cy5**, **mono-NH800CW** at a dose of 1 μmol kg^-1^, or PBS. (B) H&E staining microscopy images of heart, liver, spleen, lung, kidney of mice which intravenously injected with **ss-diNH800CW** and **mono-NH800CW**. Scale bar = 50 μm.

For biocompatibility studies, ss-didyes were intravenously injected into healthy mice. Even 14 days after injection, the mice showed no apparent body weight loss (Figure 8A), indicating good biocompatibility of all disulfide dicyanine dyes. Besides, mice were sacrificed, and main organs (heart, liver, spleen, lung, and kidney) were excised for H&E staining to evaluate the biocompatibility of various ss-didyes. No obvious damages were observed in the slices of major organs, which also suggested the excellent biocompatibility of disulfide dicyanine dyes (Figure 8B).

## Conclusion

In summary, two disulfide dicyanines were successfully synthesized, and their structures were characterized by NMR and MALDI-TOF MS. The optical properties showed that the dicyanines suffer from severe aggregation, which leads to fluorescence quenching and low brightness of probes for fluorescence imaging. However, in the redox environment of the GSH solution, the fluorescent signals of disulfide dicyanine dyes were enhanced by 27-fold. The studies on cellular uptake and tumor-bearing mice demonstrated that the disulfide bond of the disulfide dicyanines could be cleaved in cancer cells and tumor tissues and produced strong fluorescence signals. The results revealed that disulfide dicyanines dyes could provide an exciting platform for GSH triggered tumor-specific fluorescence imaging with improved T/B value. Besides, the targeted modification of disulfide dicyanine dyes is under progress to improve their targeting ability and imaging sensitivity.

## Abbreviations

GSH: glutathione
SNR: signal to noise ratio
ACQ: aggregation caused Quenching
NIR: near infrared
NMR: nuclear magnetic resonance
TSTU: O-(N-Succinimidyl)-1,1,3,3-tetramethyluronium tetrafluoroborate
DIPEA: *N,N*-diisopropylethylamine
UV: Ultraviolet
MALDI-TOF: Matrix-Assisted Laser Desorption/ Ionization Time of Flight
MS: Mass Spectrometry
T/B: Target to background
PBS: phosphate buffer saline
