# Supplementary material for: Glutathione-responsive disassembly of disulfide dicyanine for tumor imaging with reduction in background signal intensity
Source: Theranostics. 2020 Jan 12;10(5):2130–40. doi: 10.7150/thno.39673 (PMC7019170; doi:10.7150/thno.39673)
Supplement: Supplementary file 1 — Supplementary figures. [file thnov10p2130s1.pdf]

Supplementary Material

for

Glutathione-responsive disassembly of disulfide dicyanine for tumor imaging with reduction in background signal intensity

Shanyan Mo*[1,2], Xiaoting Zhang*[1], Sadaf Hameed*[1], Yiming Zhou[1], Zhifei Dai✉[1]

1 Department of Biomedical Engineering, College of Engineering, Peking University, Beijing 100871, China
2 College of Life Science and Bioengineering, Beijing University of Technology, Beijing 100124, China
Corresponding author: Professor Zhifei Dai

E-mail: zhifei.dai@pku.edu.cn

* These authors contributed equally to this work.

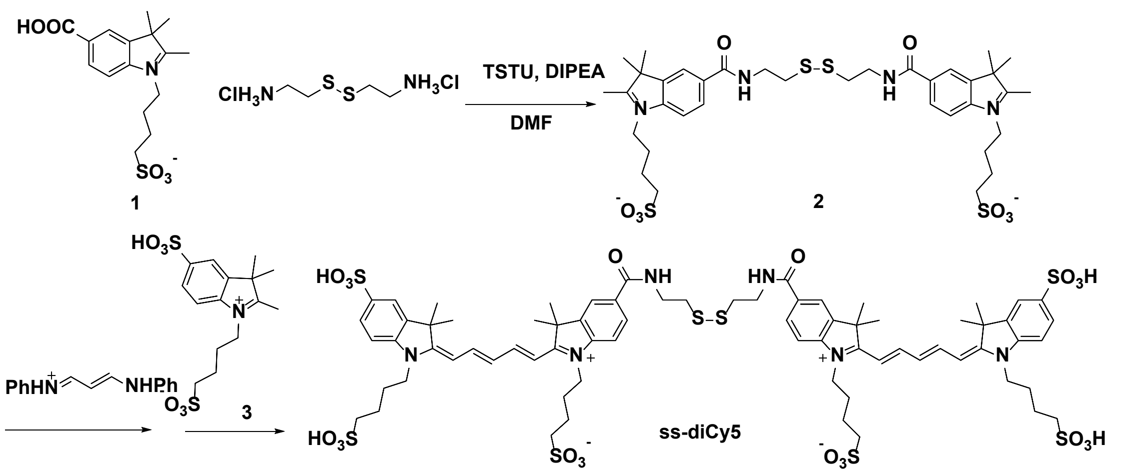

Figure S1: The synthesis route of **ss-diCy5**

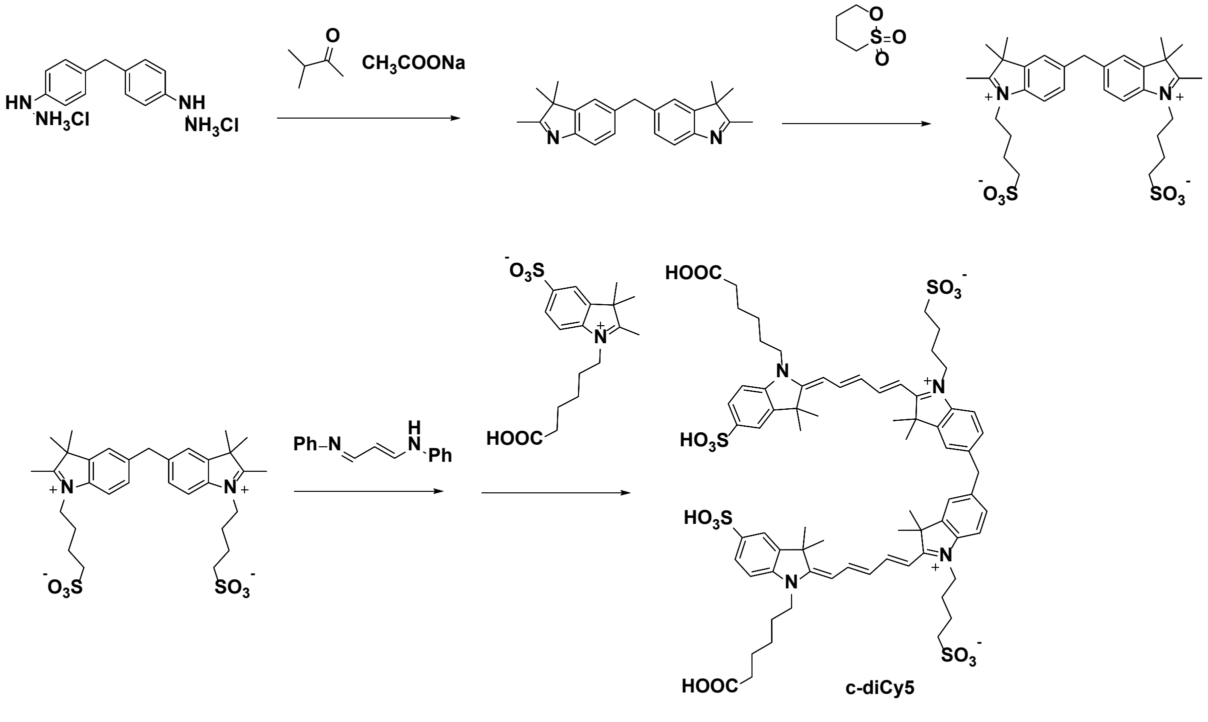

Figure S2: The synthesis route of **c-diCy5**

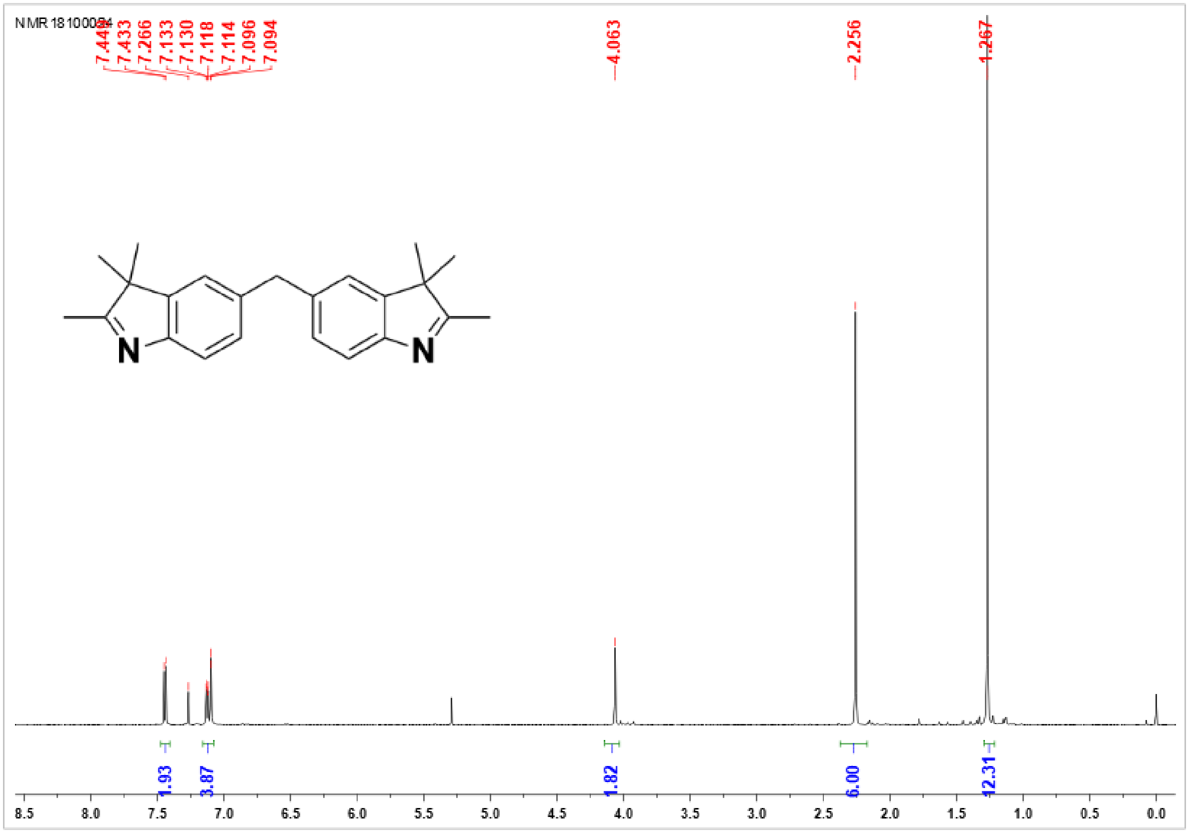

Figure S3: ^{1}H NMR spectra of diindole intermediates.

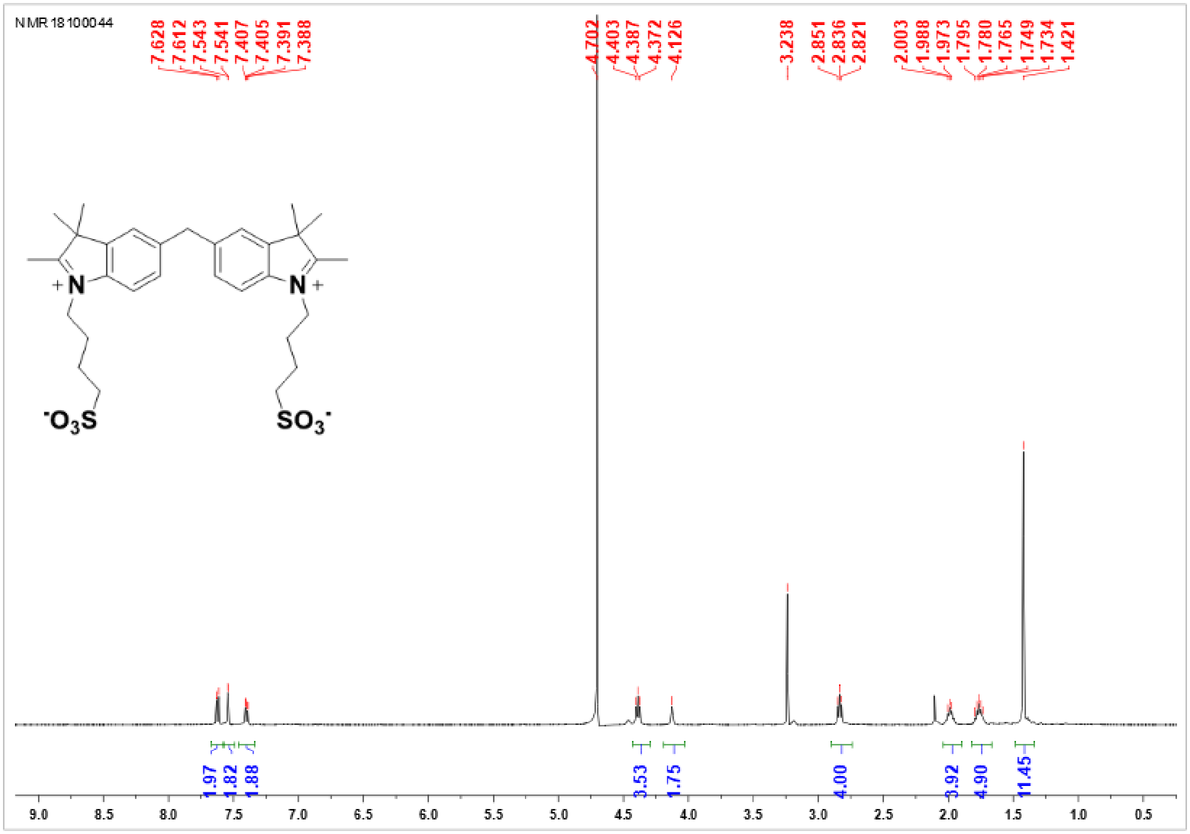

Figure S4: ^{1}H NMR spectra of Methylene diindole quaternary ammonium salt

Figure S5: HRMS spectra of **c-diCy5**

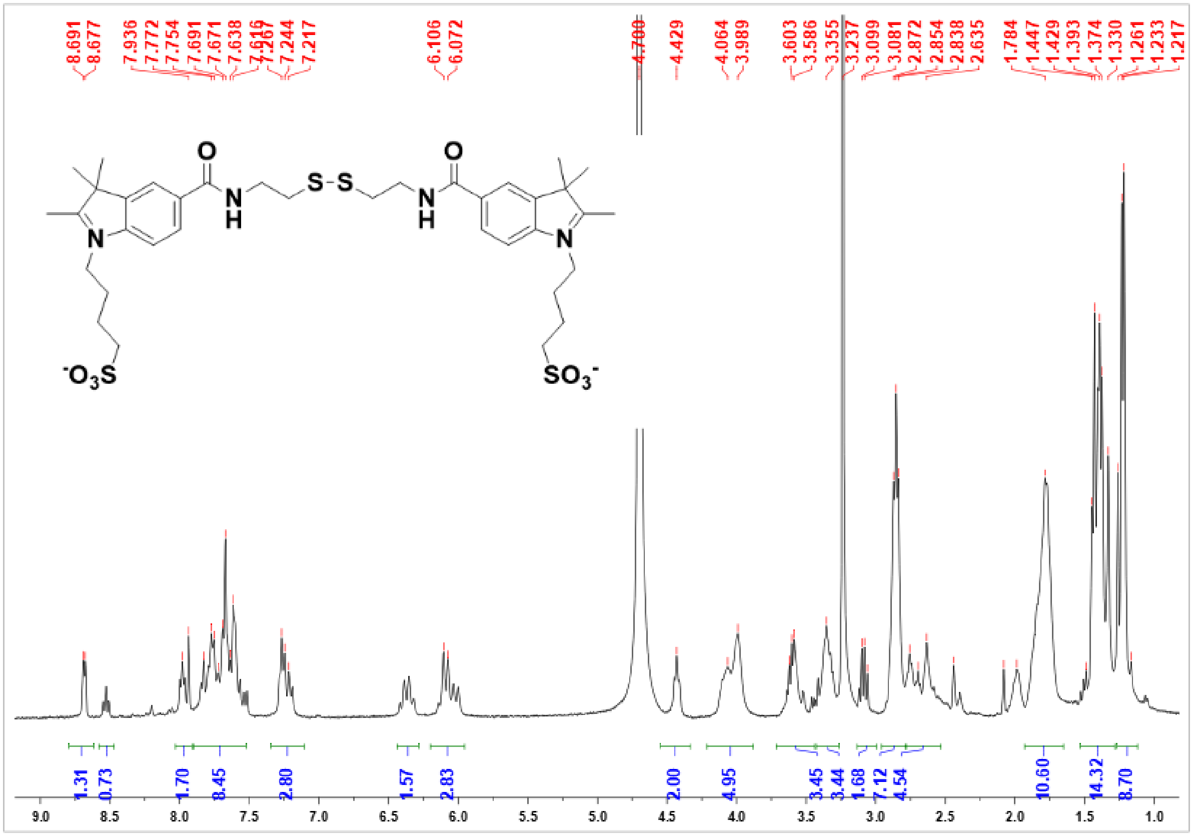

Figure S6: ^{1}H NMR spectra of disulfide diindole quaternary ammonium salt

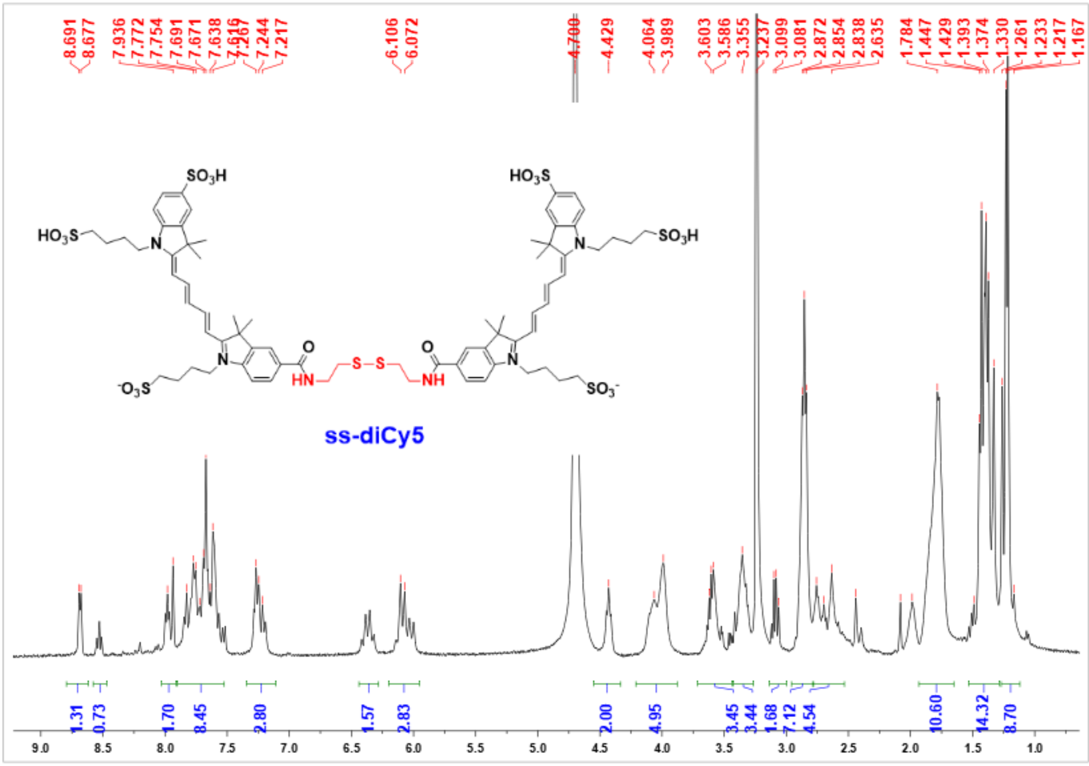

Figure S7: ^{1}H NMR spectra of **ss-diCy5**

Peking University Mass Spectrometry Sample Analysis Report

Analysis Info

Analysis Name FTICR-19060035_0_L21_000001.d

Sample ss-Cy5

Comment

Acquisition Date 7/4/2019 4:38:54 PM

Instrument Bruker Solarix XR FTMS

Operator Peking University

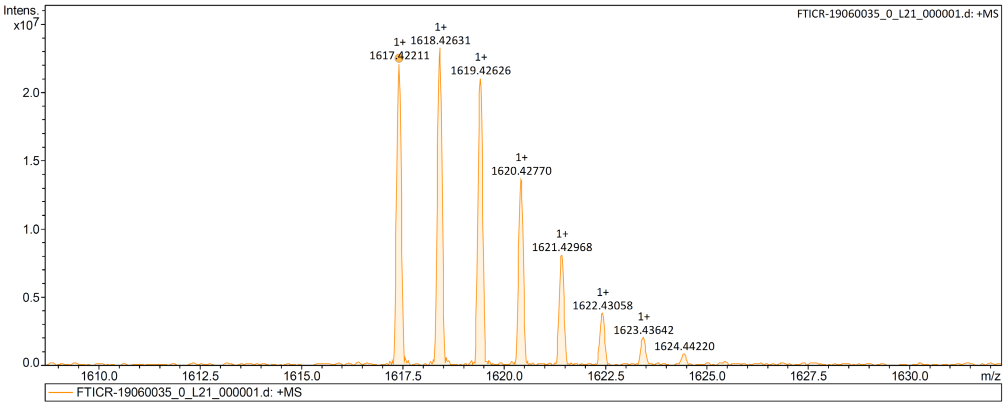

Meas. m/z	#	Ion Formula	Score	m/z	err [ppm]	Mean err [ppm]	mSigma	rdb	e⁻ Conf	N-Rule
1617.422111	1	C72H93N6O20S8	100.00	1617.420485	-1.0	-2.4	89.0	46.0	even	ok

Figure S8: HRMS spectra of **ss-diCy5**

Synthesis of ss-diNH800cw

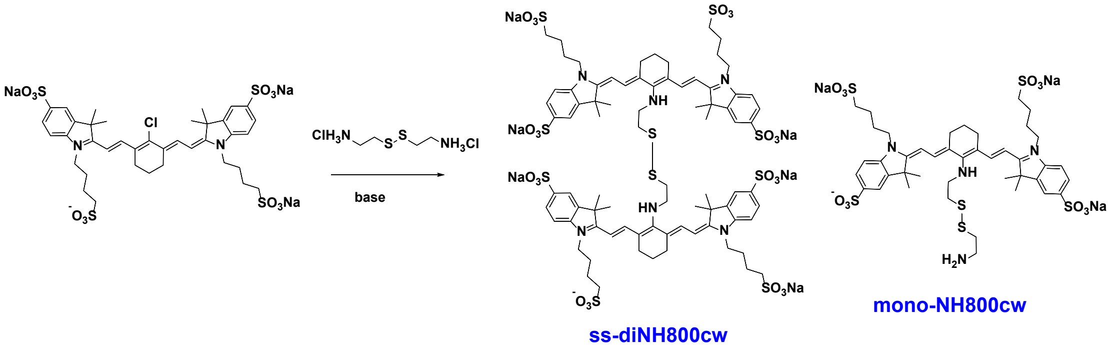

Figure S9: Synthetic route of **ss-diNH800CW**

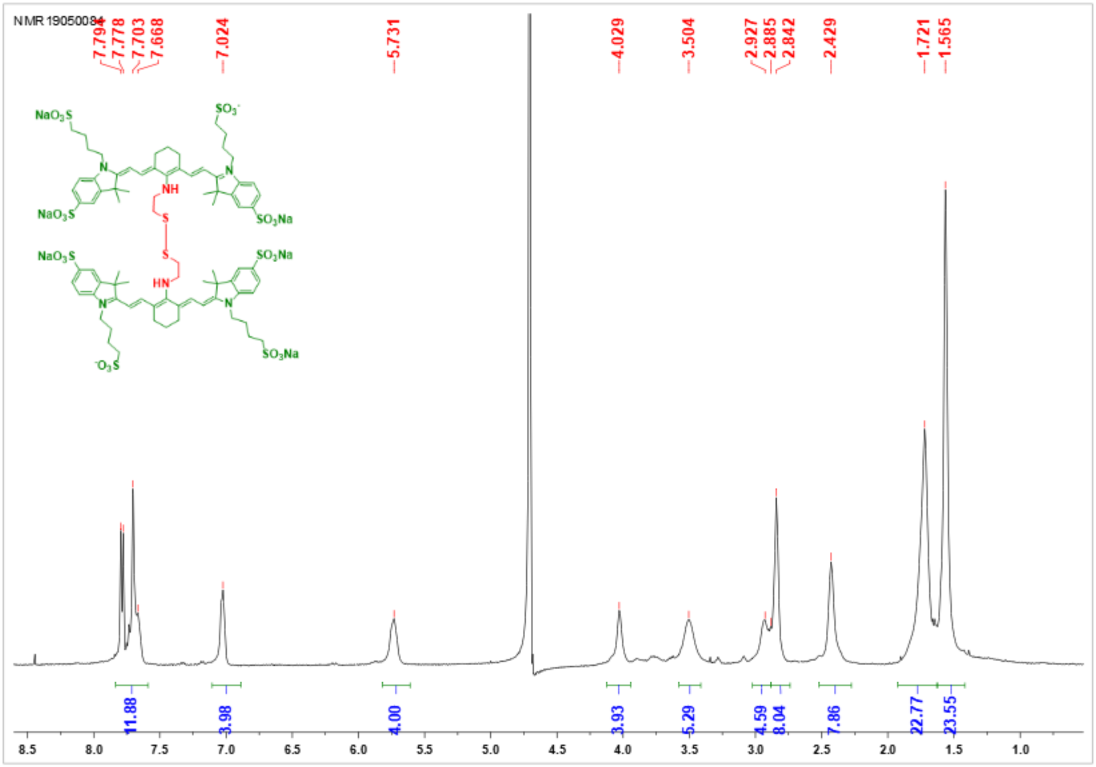

Figure S10: ^{1}H NMR spectra of **ss-diNH800CW**

Peking University Mass Spectrometry Sample Analysis Report

Analysis Info

Analysis Name	FTICR-19060032_0_L15_000002.d	Acquisition Date	7/4/2019 4:12:03 PM
Sample	ss-diNH800CW	Instrument	Bruker Solarix XR FTMS
Comment		Operator	Peking University

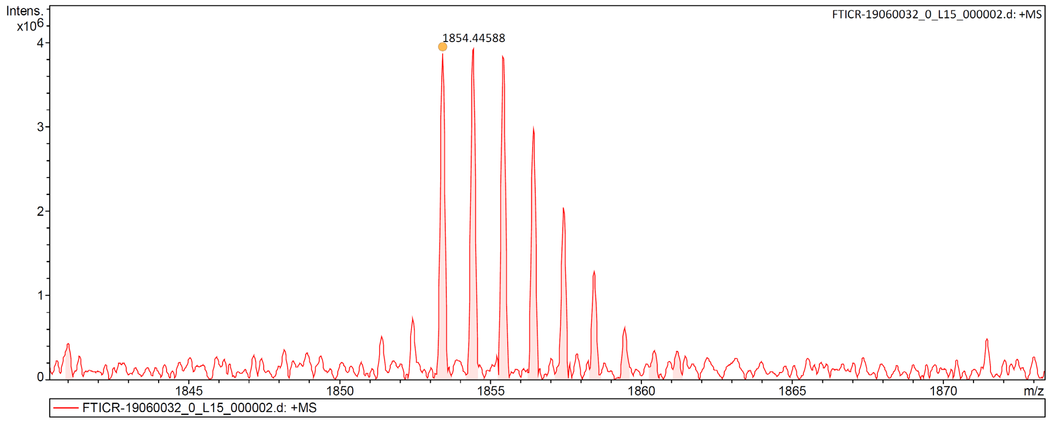

Meas. m/z	#	Ion Formula	Score	m/z	err [ppm]	Mean err [ppm]	mSigma	rdb	e$^-$ Conf	N-Rule
1853.440225	1	C80H105N6O24S10	100.00	1853.438186	-1.1	-2.5	98.5	52.0	even	ok

Figure S11: HRMS spectra of **ss- diNH800CW**

Peking University Mass Spectrometry Sample Analysis Report

Analysis Info

Analysis Name	FTICR-19060033_0_L17_000001.d	Acquisition Date	7/4/2019 4:17:20 PM
Sample	ss-monoNH800CW	Instrument	Bruker Solarix XR FTMS
Comment		Operator	Peking University

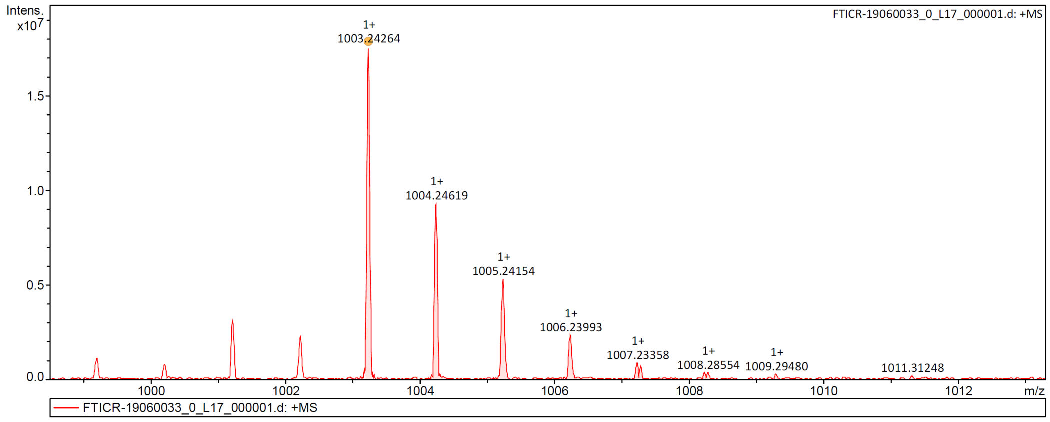

Meas. m/z	#	Ion Formula	Score	m/z	err [ppm]	Mean err [ppm]	mSigma	rdb	e$^-$ Conf	N-Rule
1003.242641	1	C42H59N4O12S6	100.00	1003.244827	2.2	6.4	51.6	28.0	even	ok

Figure S12: HRMS spectra of **mono-NH800CW**

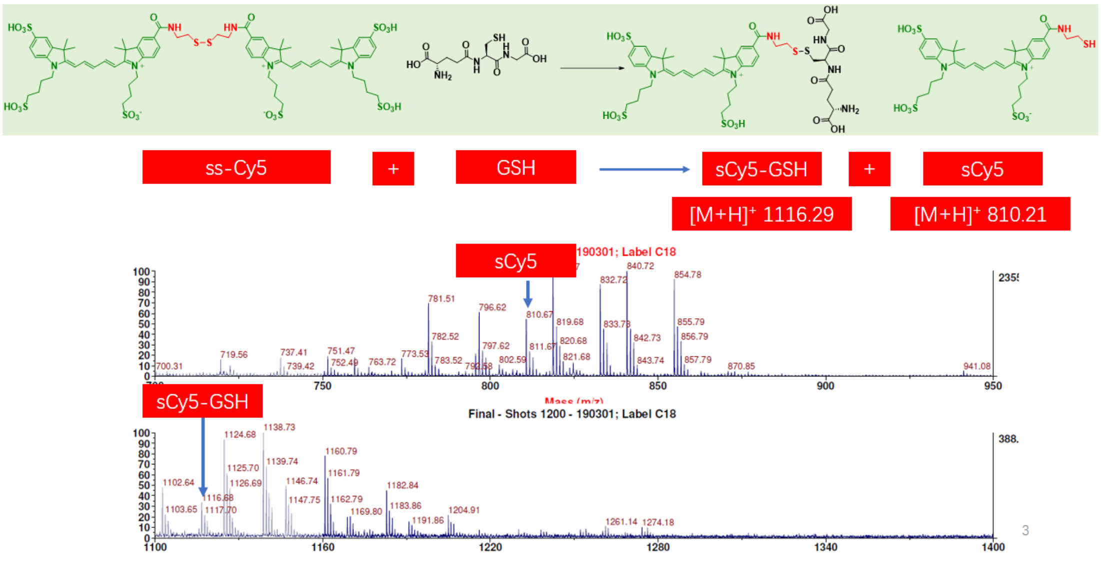

Figure S13: MALDI-TOF spectra of **ss-diCy5** in GSH solution

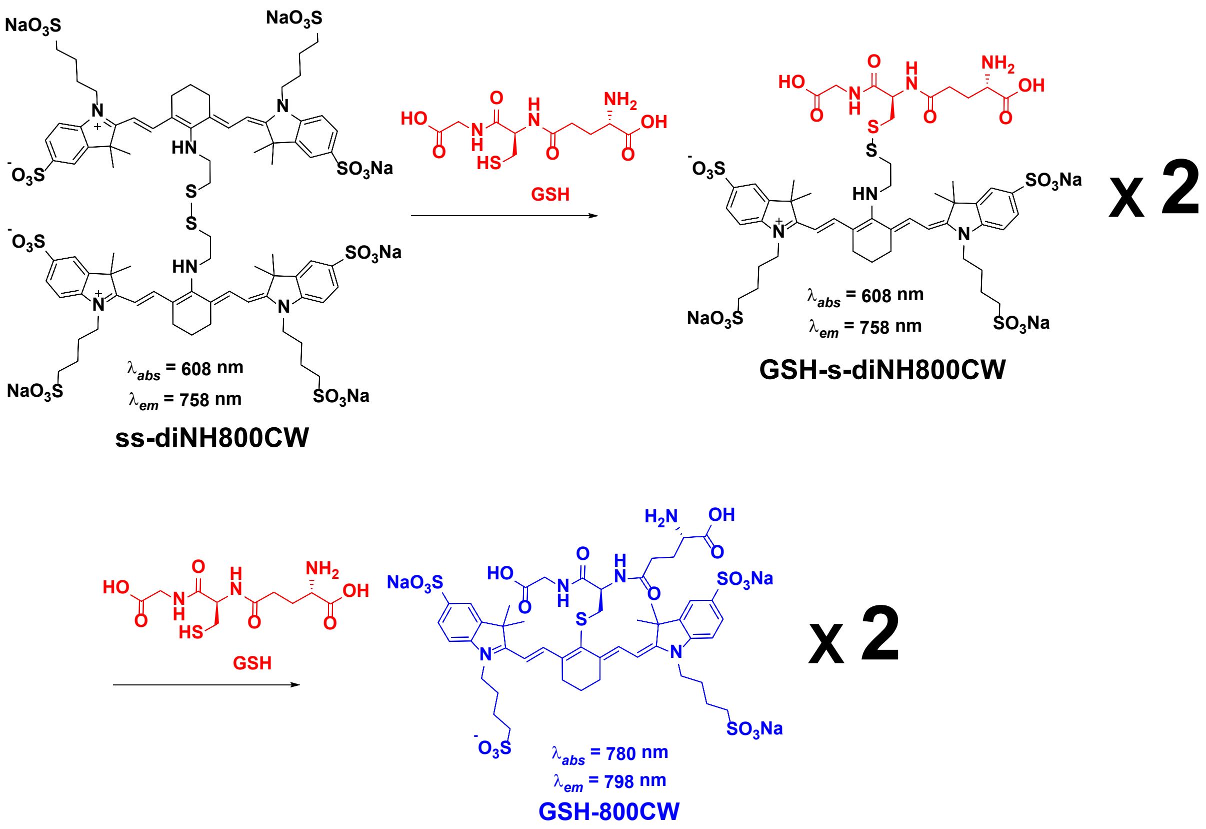

Figure S14: The proposed reactions in **ss- diNH800CW** GSH solution

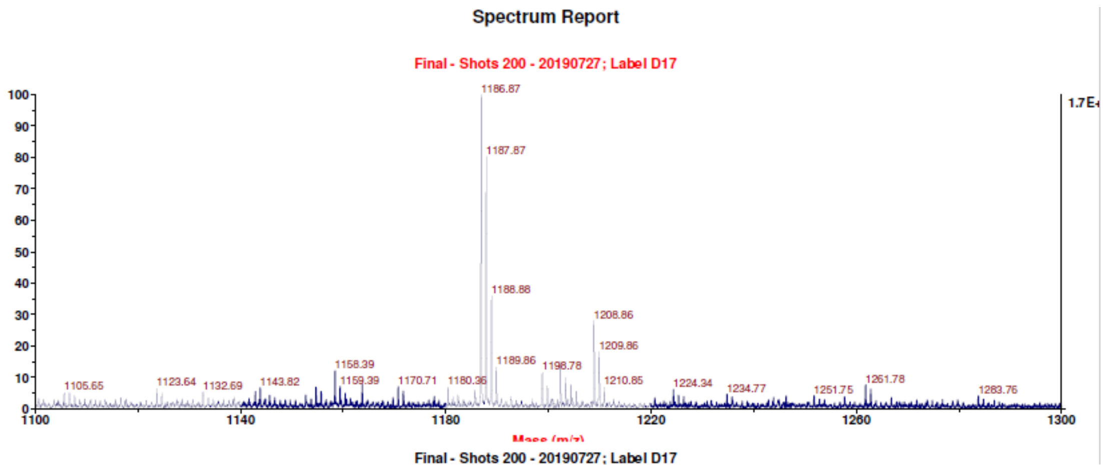

Figure S15: MALDI-TOF spectra of **ss- diNH800CW** in the GSH solution

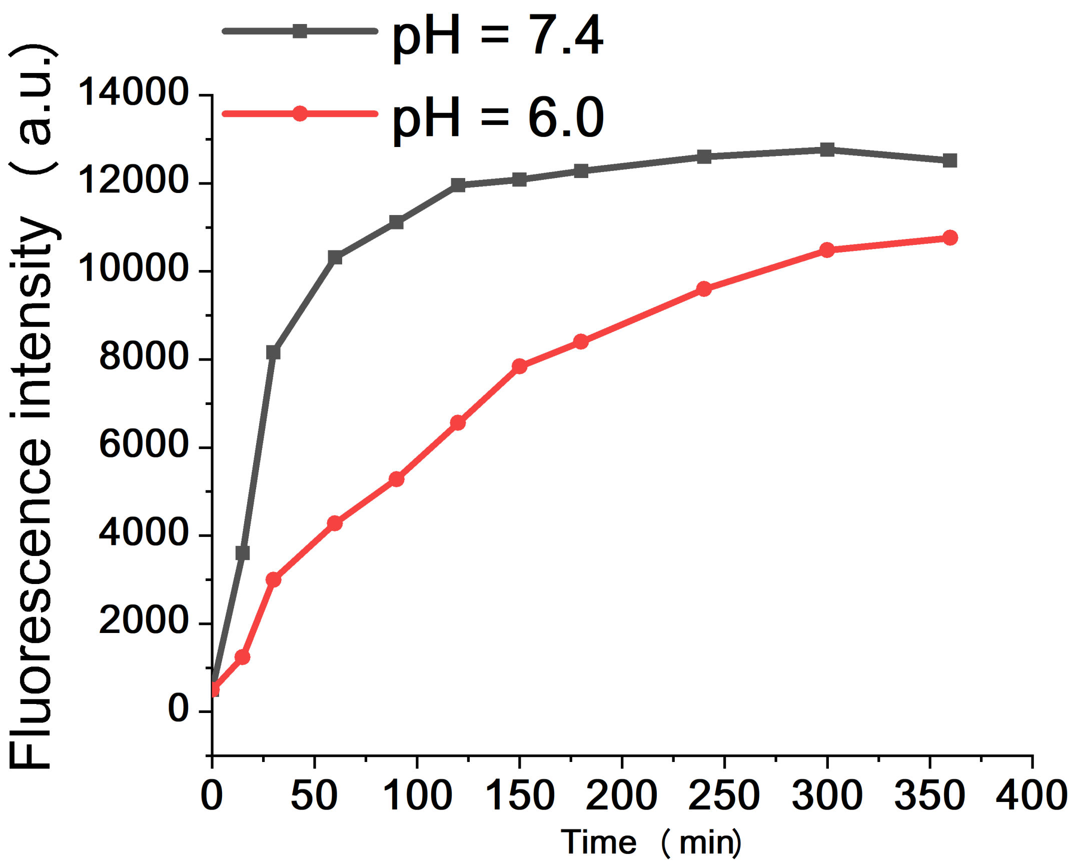

Figure S16: The fluorescence intensity enhancement of **ss-diNH800CW**(5 μM) at ~~the~~ a pH of 6.0 and 7.4 in the presence of GSH (1.5 mM) in PBS solution (25 °C). λ_{ex} = 650 nm.
